# Overcoming resistance to HER2-targeted therapy with a novel HER2/CD3 bispecific antibody

**DOI:** 10.1080/2162402X.2016.1267891

**Published:** 2017-03-10

**Authors:** Andres Lopez-Albaitero, Hong Xu, Hongfen Guo, Linlin Wang, Zhihao Wu, Hoa Tran, Sarat Chandarlapaty, Maurizio Scaltriti, Yelena Janjigian, Elisa de Stanchina, Nai-Kong V. Cheung

**Affiliations:** a Department of Surgery, Memorial Sloan Kettering Cancer Center, New York, NY, USA; b Department of Pediatrics, Memorial Sloan Kettering Cancer Center, New York, NY, USA; c Department of Medicine, Memorial Sloan Kettering Cancer Center, New York, NY, USA; d Department of Pathology, Memorial Sloan Kettering Cancer Center, New York, NY, USA; e Human Oncology & Pathogenesis Program, Memorial Sloan Kettering Cancer Center, New York, NY, USA; f Antitumor Assessment Core Facility, Memorial Sloan Kettering Cancer Center, New York, NY, USA

**Keywords:** Bispecific antibody, CD3, HER2, immunotherapy, T cells

## Abstract

T-cell-based therapies have emerged as one of the most clinically effective ways to target solid and non-solid tumors. HER2 is responsible for the oncogenesis and treatment resistance of several human solid tumors. As a member of the HER family of tyrosine kinase receptors, its over-activity confers unfavorable clinical outcome. Targeted therapies directed at this receptor have achieved responses, although development of resistance is common. We explored a novel HER2/CD3 bispecific antibody (HER2-BsAb) platform that while preserving the anti-proliferative effects of trastuzumab, it recruits and activates non-specific circulating T-cells, promoting T cell tumor infiltration and ablating HER2(+) tumors, even when these are resistant to standard HER2-targeted therapies. Its *in vitro* tumor cytotoxicity, when expressed as EC_50_, correlated with the surface HER2 expression in a large panel of human tumor cell lines, irrespective of lineage or tumor type. HER2-BsAb-mediated cytotoxicity was relatively insensitive to PD-1/PD-L1 immune checkpoint inhibition. In four separate humanized mouse models of human breast cancer and ovarian cancer cell line xenografts, as well as human breast cancer and gastric cancer patient-derived xenografts (PDXs), HER2-BsAb was highly effective in promoting T cell infiltration and suppressing tumor growth when used in the presence of human peripheral blood mononuclear cells (PBMC) or activated T cells (ATC). The *in vivo* and *in vitro* antitumor properties of this BsAb support its further clinical development as a cancer immunotherapeutic.

## Introduction

Trastuzumab has significantly improved patient outcomes in breast cancer, and has also been a key in the design and implementation of other targeted therapies.^1^ However, HER2 expression does not guarantee a clinical response to trastuzumab or other HER2-targeted therapies.^2,3^ HER2-positive breast cancer patients with metastatic disease initially respond to trastuzumab and/or other HER2-targeted therapies, but almost all eventually will develop resistance and relapse.^4^ In osteosarcoma and Ewing's sarcoma, where high levels of HER2 expression was associated with decreased survival,^5^ trastuzumab has not shown any benefit even when used in conjunction with cytotoxic chemotherapy.^6^ Furthermore, trastuzumab, like other HER-targeted therapies, has shown modest or no benefit against HER2(+) positive head and neck cancer.^7^

The reasons for these failures are complex and only partially understood. The genomic diversity and constant evolution of malignancies make them less prone to oncogene addiction, a requirement for the success of targeted therapy. Furthermore, even when oncogene addiction is present, resistance can emerge from selection pressure induced by the use of targeted therapies.^3^ In fact, despite the initial enthusiasm received, the majority of targeted therapies have not produced a significant benefit in the overall cure of patients receiving them.^8^ A different approach, one that selectively targets malignant cells that overexpress HER family receptors, and that can generate cytotoxic antitumor responses independently of the receptor activation status could be beneficial.

Redirecting the immune system against tumor cells has gained acceptance as an effective strategy to overcome resistance to cytotoxic chemotherapy and targeted therapy. In the forefront of these treatments, T-cell-based therapies constitute the most promising approach. Both T-cell-engaging bispecific antibodies (BsAb) and immune checkpoint antibody blockade have received accelerated approval from the FDA based on their outstanding clinical performance.^9^ The clinical success of chimeric antigen receptor (CAR) gene-modified T cells against non-solid tumors has further added to the enthusiasm among scientists, clinicians and the pharmaceutical industry.

The outstanding clinical responses seen with these therapies, have consolidated T cells as the most powerful effector cells within the immune system to eradicate tumor cells.^10^ Thus, several approaches that redirect them against tumor cells have been proposed and tested by many investigators. In this regard, BsAb with specificity for T cells and for tumor antigens have attracted the attention of researchers and big pharma. BsAb, in opposition to other antibody-based therapies, only require expression of their target of interest to be effective. By recruiting polyclonal T cells through the CD3 surface receptor, BsAb activate T cells irrespective of their lineage, antigen specificity, maturation, HLA restriction or co-stimulatory receptors. The direct activation of T cells, bypassing the classic T cell receptor (TCR), removes the limitations imposed by HLA restriction and its level of expression,^11^ a well-established immune resistance mechanism.^12^

Blinatumomab—a CD19/CD3 BsAb was approved in 2014 for treating acute lymphoplastic leukemia.^13^ However, despite its promising results, the unfavorable PK of these small size molecules necessitates prolonged infusions, complicating their administration.^14,15^ Furthermore, the resulting cytokine release syndrome (CRS) still poses costly and often life-threatening complications. Importantly, despite the ability of BsAb to activate T-cells, the same inhibitory pathways that regulate classic T cell function might still limit their effectiveness. For example, the heterodimeric design of a monovalent-binding HER2/CD3 bispecific antibody was inhibited by the PD-1/PD-L1 inhibitory axis.^16^

We now report a BsAb against the HER2 tumor antigen that offers two distinct advantages over the existing technologies: (1) it is based on the fully humanized HER2-specific IgG1 trastuzumab, preserving its pharmacologic advantages^17^ and bivalent binding to HER2, maximizing tumor avidity; (2) its binding to CD3 is functionally monovalent through the scFv derived from the humanized huOKT3 IgG1 sequence. Thus, HER2-BsAb is built on two mAbs with an extensive record of clinical safety. Previous studies have also shown that scFv linked to the carboxyl end of the light chain did not affect the targeting ability of these IgG forms.^18,19^ Furthermore, this is a platform with its Fc function silenced to reduce the CRS. We present data to show that this HER2-BsAb has potent antitumor properties both *in vitro* and *in vivo*, against tumor cells that are resistant to HER2-targeted therapy or to trastuzumab.

## Results

### HER2-BsAb

HER2-BsAb was designed using the same IgG-scFv format as hu3F8-BsAb,^20^ only replacing variable regions of hu3F8 with those of trastuzumab. The heavy chain was the standard human IgG1, except for the N297A mutation in the Fc region to remove glycosylation. The light chain was constructed by extending the trastuzumab IgG1 light chain with a C-terminal (G_4_S)_3_ linker followed by huOKT3 scFv.^20^ The DNAs encoding both heavy chain and light chain were inserted into a mammalian expression vector, transfected into CHO-S cells, and stable clones of highest expression were selected. Supernatants were collected from shaker flasks and purified on protein A affinity chromatography.^20^

The SEC-HPLC and SDS-PAGE of the HER2-BsAb was analyzed. Under reducing SDS-PAGE conditions, HER2-BsAb gave rise to two bands at around 50 KDa, since the huOKT3 scFv fusion to trastuzumab light chain increased the MW to ∼50 KDa (data not shown). SEC-HPLC showed a major peak (97% by UV analysis) with an approximate MW of 210 KDa, as well as a minor peak of multimers (data not shown). The BsAb remained stable by SDS-PAGE and SEC-HPLC after multiple freeze and thaw cycles (data not shown).

### HER2-BsAb retained specificity, affinity and anti-proliferative effects of trastuzumab

**Figure 1.**
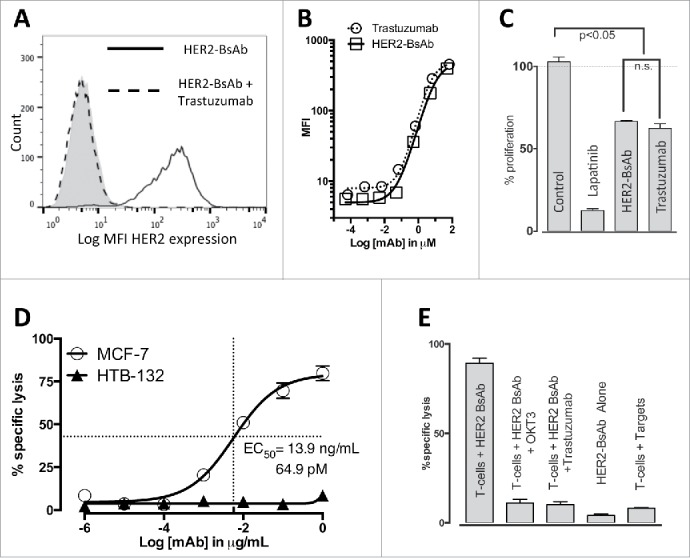
*In vitro* characterization of HER2-BsAb. (A) HER2-BsAb has the same specificity as trastuzumab. Pre-Incubation of the HER2(+)^high^ SKOV3 cells with trastuzumab prevents HER2-BsAb binding. (B) HER2-BsAb and trastuzumab have similar avidity for SKOV3 cells. MFI was plotted against the antibody concentration. (C) HER2-BsAb maintained same anti-proliferative effects as trastuzumab against the trastuzumab-sensitive SKBR3 cells. (D) HER2-BsAb mediates T cell cytotoxicity against the HER2(+)MCF-7 cells but not the HER2(−) HTB-132 cells. (E) Blocking of HER2 or CD3 by trastuzumab or huOKT3, abrogates HER2-BsAb T-cell cytotoxicity. HER2(+) SCCHN PCI-13 cells were used in the cytotoxicity assay. For this experiment, 0.1 ug/mL of HER2-BsAb with 10 ug/mL of the blocking antibodies was used.

To determine if HER2-BsAb retained the specificity of trastuzumab, the HER2(+)^high^ SKOV3 ovarian carcinoma cell line was pre-incubated with 10 µg/mL of trastuzumab for 30 min and then immunostained using 1 µg/mL HER2-BsAb labeled with Alexa 488 (Fig. 1A). Pre-incubation with trastuzumab prevented HER2-BsAb binding to SKOV3 cells, demonstrating that these antibodies shared the same specificity. To compare the avidity of HER2-BsAb to trastuzumab, the same cell line was incubated with 10-fold downward dilutions (from 10 µg/mL to 1 × 10^−5^ µg/mL) of trastuzumab or HER2-BsAb and analyzed by flow cytometry. The mean fluorescence intensity (MFI) was plotted against the antibody concentration in µM. Again the similarity in the binding curves confirmed that trastuzumab and HER2-BsAb had similar binding avidities for their common HER2 target (Fig. 1B).

Finally, the trastuzumab-sensitive breast carcinoma cell line SKBR3 was treated with isotype control mAb, 10 nM lapatinib (as a positive control), 10 ug/mL HER2-BsAb or 10 ug/mL trastuzumab for 72 h and cell proliferation was assayed. As shown in Fig. 1C, trastuzumab and HER2-BsAb had similar anti-proliferative effects that were significant compared with the negative control. As expected, lapatinib showed the strongest inhibition of cell proliferation.

### HER2-BsAb-redirected T cell cytotoxicity was HER2 specific and dependent on CD3

Prior to the cytotoxicity assay, HER2-BsAb was shown capable of binding different T cells at the similar level (MFI around 450 with the BsAb concentration of 1 ug/10^6^ cells), no matter whether they were naive T cells purified from fresh PBMC or activated T cells (ATCs) (Fig. S1A). To establish the specificity of cytotoxic responses by T cells in the presence of HER2-BsAb, HER2(−) breast carcinoma HTB-132 cells and HER2(+) MCF-7 cells were tested in a cytotoxicity assays using ATCs (E:T ratio of 10:1) and HER2-BsAb at decreasing concentrations (Fig. 1D). Cytotoxicity was robust for HER2(+) cells but absent for HER2(−) cells. In fact, HER2-BsAb was able to redirect efficient T cell killing no matter whether BsAb was present throughout the 4 h assay (mixing), or used to pre-arm T cells and then washed off, or to pre-target AU565 tumor cells and then washed off. Although pre-targeted AU565 cells were killed as well as mixing all three together, pre-armed T cells were less potent due to the low avidity of BsAb binding to CD3 on T cells (Fig. S1B). To demonstrate the dependency of cytotoxicity on both HER2 and CD3, HER2-BsAb cytotoxicity against HER2(+) SCCHN cell line PCI-13 was tested in the presence of trastuzumab, or the CD3-specific blocking huOKT3 IgG1 (Fig. 1E). Pre-incubation with either trastuzumab or huOKT3 prevented HER2-BsAb-mediated T cell cytotoxicity.

### HER2-BsAb-mediated cytotoxicity against HER2(+) cell lines that were resistant to other HER2-targeted therapies

**Figure 2.**
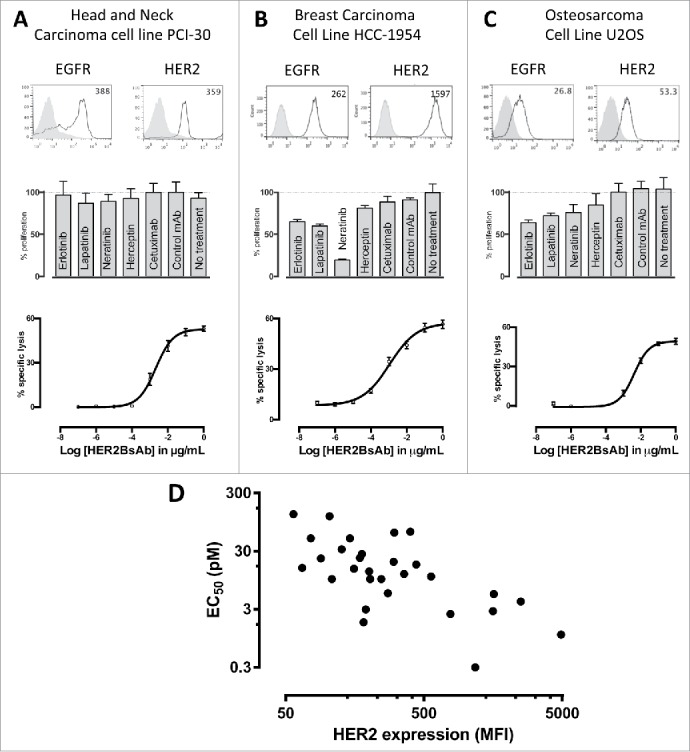
HER2-BsAb mediates cytotoxic responses against carcinoma cell lines resistant to other HER-targeted therapies. Three representative cell lines were used for FACS assay (upper panel), proliferation assay (middle panel) and HER2-BsAb-mediated CTL assay (lower panel): (A) SCCHN PCI-30, (B) breast carcinoma HCC-1954 and (C) osteosarcoma U2OS. (D) HER2-BsAb EC50 inversely correlates with level of HER2 expression. Each of the cell lines used in a cytotoxicity assay (Table 1) was assayed at least twice. The EC50 was determined each time and averaged. These values (except those beyond assay limit 5 nM) were compared with HER2 expression (MFI).

A panel of total 39 cell lines from different tumor systems (breast, ovarian, gastric, head and neck, sarcoma, etc.) was characterized for their HER2 expression level by flow cytometry and CTL activity (Table 1). In this panel, 75% of these cells were tested positive for HER2 expression. Representative cell lines were assayed for their sensitivity to tyrosine kinase inhibitors (10 nM each of Erlotinib, Lapatinib, Neratinib), or HER antibodies (10 ug/mL each of trastuzumab and cetuximab), as well as HER2-BsAb-mediated T-cell cytotoxicity. Figs. 2A–C showed three representative lines from three different tumor systems. As shown, HER2 expression, even in low quantities, was sufficient to mediate T cell cytotoxicity in the presence of ATC and HER2-BsAb in cell lines otherwise resistant *in vitro* to HER-targeted therapies. When these cell lines were tested for cytotoxicity in the presence of ATC and HER2-BsAb, sensitivity to HER2-BsAb expressed as EC50, inversely correlated with surface HER2 expression in general (Fig. 2D, Table 1).

**Table 1.** HER2 Expression and EC50 in the presence of ATC and HER2-BsAb in 39 different cell lines from different tumor systems.

Tumor type	Cell line	HER2 expression (MFI)^*^	EC50 (pM)^**^
Breast carcinoma	AU565	1,175	0.3
Gastric carcinoma	NCI-N87	4,900	1.1
Ovarian carcinoma	OVCAR3	183	1.8
Breast carcinoma	MDA-MB-361	777	2.5
Ovarian carcinoma	SKOV3	1,577	2.8
Melanoma	SKMEL28	190	3
Breast carcinoma	SKBR3	2,506	4.1
Breast carcinoma	HCC1954	1,597	5.5
Head and neck cancer	SCC90	274	5.7
Ewings	SKEAW	246	10
Osteosarcoma	CRL1427	108	10
Rhabdomyosarcoma	HTB82	204	10
Osteosarcoma	RG 160	563	11
Head and neck cancer	PCI-30	359	12.2
Gastric carcinoma	KATO III	201	13.5
Melanoma	HT-144	156	15
Neuroblastoma	NB5	66	15.5
Osteosarcoma	RG 164	439	17.7
Head and neck cancer	UM SCC47	302	19.8
Osteosarcoma	U20S	90	22.5
Gastric adenocarcinoma	AGS	172	23
Head and neck cancer	UDSCC2	178	26.9
Gastric carcinoma	SNU-16	29	30.5
Head and neck cancer	93VU147T	127	32.4
Ewings	SKES-1	146	50
Breast carcinoma	MDA-MB-231	76	50.2
Head and neck cancer	15B	305	62.8
Breast carcinoma	MCF7	398	64.9
Cervical cancer	HeLa	104	120.7
Melanoma	M14	57	130
Breast carcinoma	MDA-MB-468	6	>5,000
Neuroblastoma	NMB7	12	>5,000
Neuroblastoma	SKNBE(2)C	8	>5,000
Neuroblastoma	IMR32	6	>5,000
Neuroblastoma	SKNBE(2)S	4	>5,000
Neuroblastoma	SKNBE(1)N	3	>5,000
Small cell lung cancer	NCI-H524	14	>5,000
Small cell lung cancer	NCI-H69	10	>5,000
Small cell lung cancer	NCI-H345	6	>5,000

* FACS analysis using trastuzumab IgG1, with rituximab as negative control (MFI set at 5).

** 4 h ^51^Cr release assay at 10:1 E:T ratio. Maximum antibody concentration at 1 ug/mL. EC50 (concentration of antibody at half-maximal killing) was calculated using SigmaPlot.

### HER2-BsAb-mediated *in vitro* T cell cytotoxicity was relatively insensitive to PD-L1 expression on the tumor targets or PD-1 expression on T cells

**Figure 3.**
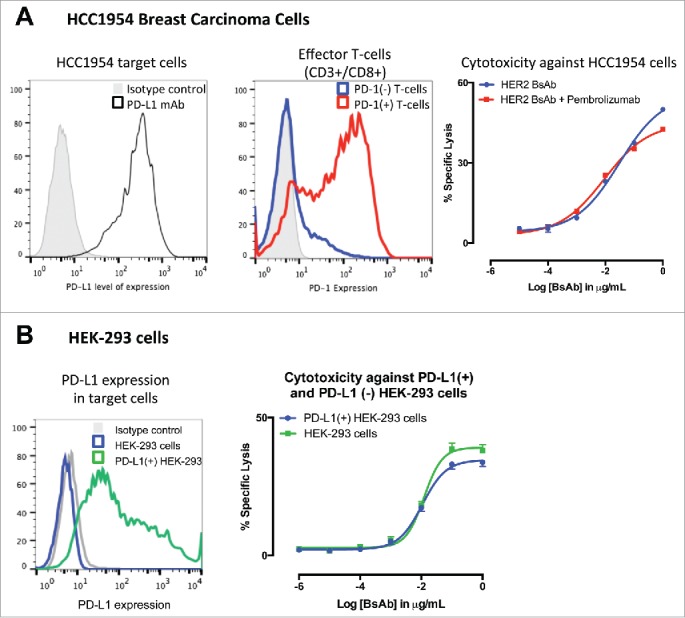
HER2-BsAb-mediated *in vitro* T cell cytotoxicity was relatively insensitive to PD-L1 expression on the tumor targets or PD-1 expression on T cells. (A) FACS analysis of PD-L1 expression in HCC1954 cells (left panel), of induced PD-1 expression in ATCs (middle panel), and HER2-BsAb-mediated cytotoxicity (right panel). (B) FACS analysis of PD-L1 expression in HEK-293 cells (left panel), and HER2-BsAb-mediated cytotoxicity using the ATCs as in (A) (middle panel). Mean + SEM (n = 6).

Activation of tumor-specific CTL in the tumor microenvironment is known to promote expression PD-1/PD-L1 leading to T cell exhaustion or suppression, a phenomenon termed “adaptive immune resistance”.^21^ The presence of PD-1/PD-L1 pathway has also been reported to limit the antitumor effects of T-cell-engaging BsAb.^16^ To determine if HER2-BsAb had this same limitations, PD-1(+) ATCs were used against the HER2(+) PD-L1(+) breast carcinoma cell line HCC1954 with or without the PD-1-specific antibody pembrolizumab. As shown in Fig. 3A, PD-1(+) T cells generated similar cytotoxic responses in the presence of HER2-BsAb no matter whether pembrolizumab was present or not. When HER2(+) human embryonic kidney cells (HEK-293) were transfected with the full sequence of PD-L1 and used as targets, cytotoxicity against cells expressing PD-L1 was not significantly different to the one observed in non-transfected HEK-293 cells, although maximal cytotoxicity was slightly less with PD-L1(+) HEK-293 versus PD-L1(−) parental HEK-293 (Fig. 3B).

### HER2-BsAb was effective against HER2(+) xenografts

**Figure 4.**
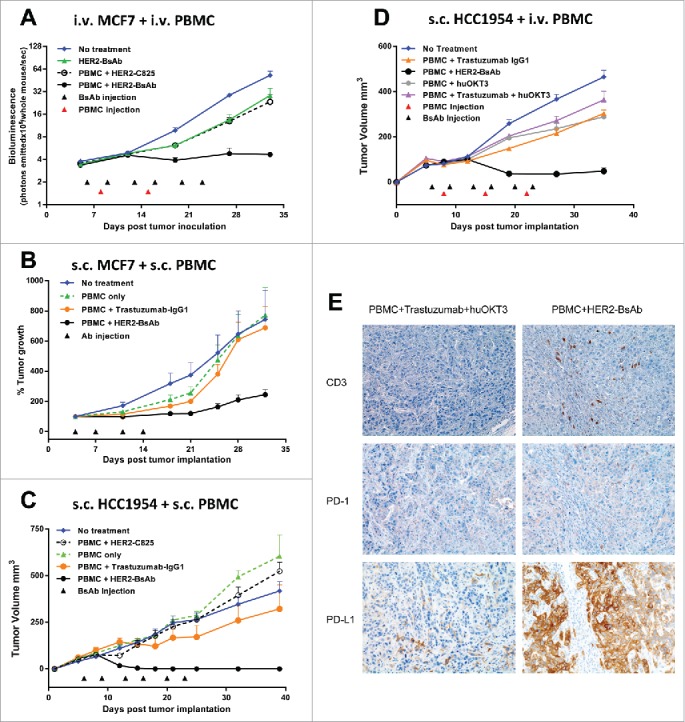
HER2-BsAb is effective against HER2(+) breast cancer cell line xenografts. Treatment schedules were marked on the figures, and doses of BsAbs and effector cells were detailed in the Results. Data shown as mean + SEM (n = 5). (A) *iv tumor plus iv effector cells model*: Bioluminescence changes of MCF7 breast cancers during treatment. (B, C) *sc tumor plus sc effector cells (mixing) model*: % tumor growth of MCF7 (B), and tumor volume changes of HCC1954 (C). (D) *sc tumor plus iv effector cells model*: tumor volume changes of HCC1954. (E) HCC1954 sc tumor model as in (D), with treatments of one dose of PBMC (2×10^7^ cells iv) at day 14, and two doses of BsAbs (100 ug iv) at day 12 and 15. Representative images (200× magnifications) of IHC staining of tumor sections collected 5 d after iv PBMC were shown.

**Figure 5.**
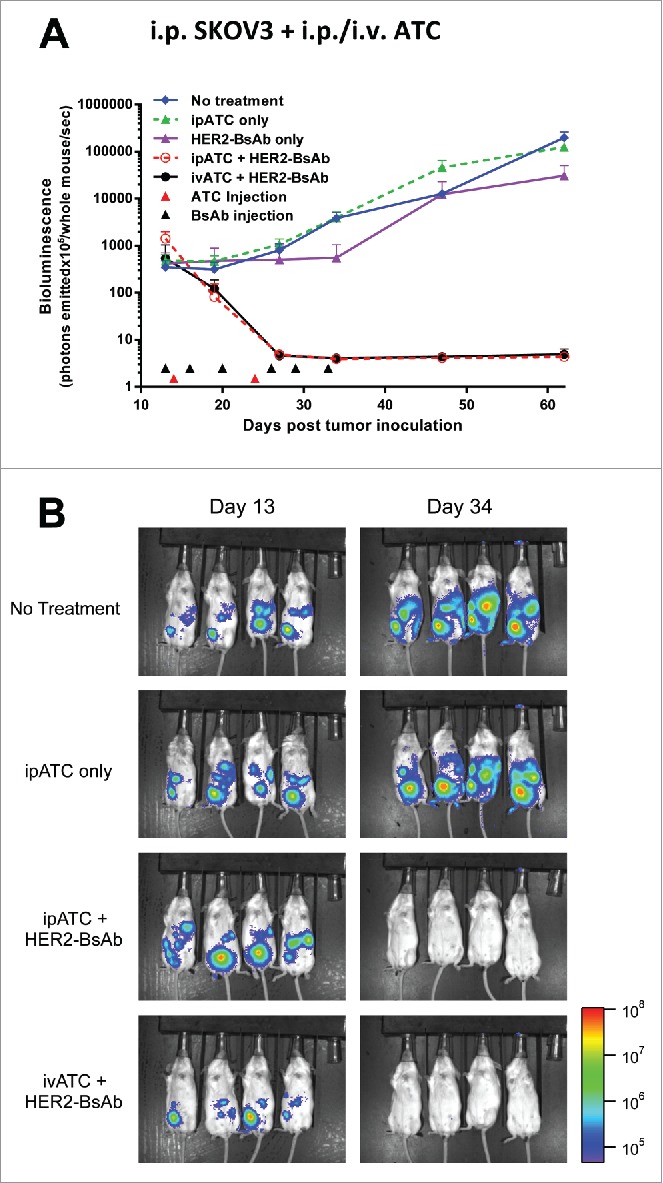
HER2-BsAb is effective against HER2(+) ovarian cancer cell line xenografts. Treatment schedules were marked on the figures, and doses of BsAbs and effector cells were detailed in the Results. Data shown as mean + SEM (n = 4). (A) *ip tumor plus ip/iv effector cells model*: Bioluminescence changes of SKOV3-luc ovarian cancers during treatment. (B) Representative bioluminescence images at the beginning (Day 13) and ending (Day 34) of the treatment were shown.

**Figure 6.**
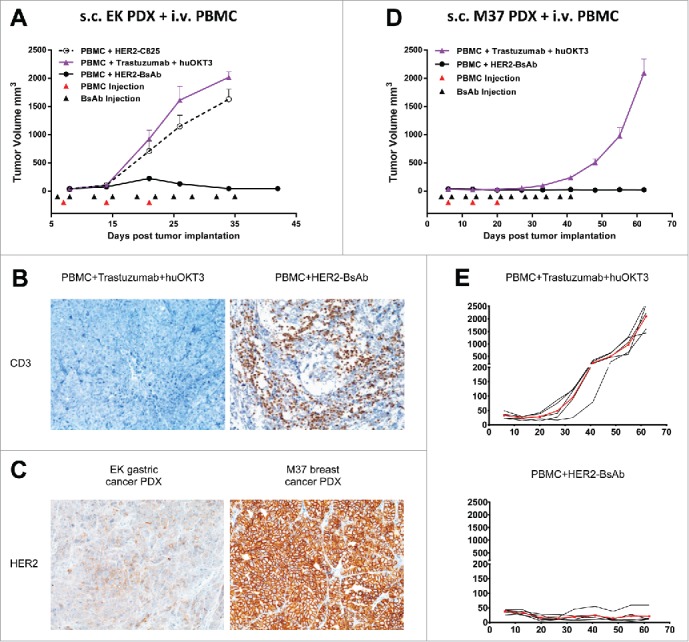
HER2-BsAb is effective against HER2(+) PDXs. sc tumor plus iv effector cells model was used for PDXs. Treatment schedules were marked on the figures, and doses of BsAbs and effector cells were detailed in the Results. Data shown as mean + SEM (n = 5). (A) Tumor volume changes of EK gastric cancer PDX. (B) IHC images of CD3 staining from another experiment with similar setting as in (A). Representative images (200× magnifications) of IHC staining of tumor sections collected 36 d after iv PBMC were shown. (C) IHC images (200× magnifications) of HER2 staining of control treated tumor sections. (D, E) Average tumor volume changes of M37 breast cancer PDX (D), and tumor growth of five individual mouse (black thin line) and averages (red thick line) in each group (E).

To determine the *in vivo* efficacy of HER2-BsAb, the breast carcinoma cell lines HCC1954 (HER2^high^), MCF-7 (HER2^low^) and ovarian carcinoma cell line SKOV3, as well as HER2(+) patient derived breast cancer and gastric cancer xenografts (PDXs) were used in DKO mice xenograft models. Four tumor models differing in tumor locations and effector routes were used, with the first three described before^20^ to simulate different clinical situations: (1) iv tumor cells/iv effector PBMC; (2) sc tumor cells/sc PBMC; (3) sc tumor cells/iv PBMC; and (4) ip tumor cells plus ip or iv effector T cells to simulate ovarian cancer metastasizing to the peritoneal cavity. Figs. 4 and 5 summarize the results of these experiments using cell lines, and Fig. 6 using PDXs (M37 breast cancer and EK gastric cancer).

3 × 10^6^ HER2^low^ MCF-7-luc (carrying luciferase reporter) cells were inoculated via tail vein i.v. injection into DKO mice. When tumor presence was confirmed by bioluminescence, mice were treated with HER2-BsAb or control BsAb (20 ug i.v., 2 ×/wk × 3 weeks), in combination with PBMC (5 × 10^6^ i.v., q wk × 2 weeks). Mice were evaluated for tumor burden using luciferin bioluminescence every week. In this hematogenous disease model, MCF-7 cells were completely eradicated without disease progression (Fig. 4A). This same cell line was implanted sc mixed with effector PBMC (1:1, 7 × 10^6^ each), and treated with HER2-BsAb (10 ug i.v., 2 ×/wk × 2 weeks in first experiment; or 20 ug i.v. 2 ×/wk × 3 weeks in second experiment). In both experiments, HER2-BsAb caused a significant delay in tumor progression, while PBMC + trastuzumab or PBMC alone were ineffective (Fig. 4B). In two other separate experiments, 5 × 10^6^ HER2(+) breast carcinoma HCC1954 cells was implanted sc mixed with 2.5 × 10^6^ PBMC (2:1). Again after either four or six injections of HER2-BsAb (20 ug i.v. per dose), there was complete suppression of tumor growth, while trastuzumab or control BsAb HER2-C825 almost had no effect (Fig. 4C). In the third model, where sc 5 × 10^6^ HCC1954 xenografts were treated with iv PBMC (5 × 10^6^, q wk × 3 weeks), and iv HER2-BsAb (100 ug, 2 ×/wk × 3 weeks), tumor growth was substantially delayed (two separate experiments), in contrast to only modest effects for PBMC + trastuzumab + huOKT3, PBMC + trastuzumab, or PBMC + huOKT3 (Fig. 4D). For HCC1954 xenografts, the following observations were made: when effector PBMCs were mixed with tumor cells sc, complete tumor regression without recurrence was seen followed past 90 d from tumor implantation. When effector PBMC was administered iv, there was significant reduction in the size of the tumors, but complete regression was only observed in a subset of animals (data not shown).

Since T cell homing into tumor is critical for antitumor response in cancer immunotherapy,^22^ T cell tumor infiltration was studied using the sc tumor model described in Fig. 4D. Tumors were collected 5 d after iv PBMC and immunohistochemistry (IHC) was performed (Fig. 4E). T cell tumor infiltration by CD3(+) staining was detected only in PBMC + HER2-BsAb-treated group but not in control group (PBMC + Trastuzumab + huOKT3). These infiltrated T cells also had PD-1 expression although very weak. Interestingly, PD-L1 expression in the tumor cells was strongly upregulated in the HER2-BsAb-treated mice, presumably induced by the cytokines released by the infiltrated T cells in the vicinity. But still, HER2-BsAb treatment eradicated these tumors. This was consistent with the *in vitro* data (Fig. 3) showing HER2-BsAb-mediated T cell cytotoxicity was relatively insensitive to or sufficient to overcome the PD-1/PD-L1 immune checkpoint inhibition.

To simulate ovarian cancer that metastasized into the peritoneal cavity, 1 × 10^5^ ovarian cancer SKOV3-luc cells were injected peritoneally in DKO mice, and treatments were started after confirming tumor growth by bioluminescence. Besides ivATC, ipATC was also tested as a source of effectors. As shown in Figs. 5A and B, after treated with ATC ((7.5 × 10^6^ iv or ip, once a week × 2 weeks), and ip HER2-BsAb (100 ug, 2 ×/wk × 3 weeks), tumors were completely eradicated without evidence of recurrence at follow-up. Both ivATC and ipATC were equally effective in this fourth model.

We next tested HER2-BsAb using PDXs, since they could approximate tumor heterogeneity and microenvironment typically found in fresh human tumor specimens. To determine whether HER2-BsAb is effective against PDXs, two HER2(+) PDXs were tested using the sc tumor cells/iv PBMC model similar to the one described in Fig. 4D. When the gastric PDX (EK) was passaged sc in DKO mice, and treated with iv PBMC (1 × 10^7^, once a week × 3 weeks), and iv HER2-BsAb (100 ug, 2 ×/wk × 5 weeks), tumors were completely eradicated without disease progression (Fig. 6A), accompanied by substantial amount of T cell tumor infiltration (Fig. 6B), even though the HER2 expression level was relatively low compared with the M37 breast cancer PDX described next (Fig. 6C). In this next experiment, M37 PDX was passaged sc in DKO mice, and treated with iv PBMC (7.5 × 10^6^, once a week × 3 weeks), and iv HER2-BsAb (100 ug, 2 ×/wk × 6 weeks). Tumor growth was completely suppressed in contrast to control group (PBMC + trastuzumab + huOKT3) (Figs. 6D and E). Taken together, these experiments showed that HER2-BsAb was effective against early passaged HER2(+) human tumor specimens.

## Discussion

We described a HER2-specific BsAb with potent T-cell-mediated antitumor activity *in vitro* and *in vivo*, ablating tumors or delaying tumor growth in four separate tumor-human PBMC compartment models. Unlike monovalent bispecifics, this HER2-BsAb had identical anti-proliferative capacity to its parental trastuzumab. Its serum half-life and AUC were similar to IgG (data not shown). Most importantly, the T-cell-mediated cytotoxicity it induced was relatively insensitive to inhibition by the PD-1/PD-L1 pathway, not described previously for this IgG-scFv platform.^20^ To date, other than the anti-GD2 hu3F8-BsAb reported from our laboratory,^20^ no published T cell redirecting BsAb have used this format. The ability of this IgG-scFv antibody platform to recruit circulating lymphocytes into the tumor stroma is critical, given the importance of TIL cells for a successful antitumor effect in most checkpoint blockade studies to date,^21^ distinguishing responders from non-responders.^23^

Schreiber has proposed that tumor cells evolve to evade the immune system through a process termed “immuno-editing.” Broadly speaking, this process occurs at two levels: by changes within the (1) tumor cells or (2) the tumor microenvironment. Tumor cells can evade T cell responses by downregulating MHC/peptide complexes or by decreasing tumor-antigen expression or through the loss of antigen presenting machinery components. On the other hand, suppression of the immune response in the tumor microenvironment is the result of T-regulatory cells, myeloid-derived suppressor cells, M2 macrophages,^24-26^ immuno-suppressive cytokines (including IDO),^27^ immune checkpoint molecules^28,29^ and the consumption of IL-2.^30^

Immune checkpoint antibodies that target the CTLA-4 and PD-1/PD-L1 inhibitory pathways are capable of reversing the inhibitory tumor-microenvironment and producing significant and long-lasting clinical responses.^31^ However, these strategies are not effective against all tumor types and their success is limited to a subset of patients. Efforts to determine biomarkers that can predict outcomes of these therapies are underway. Durable clinical responses to the CTLA-4 blockade were recently correlated with tumor mutational load and the expression of antigenic tetra-peptides that resembled those found in viral and bacterial pathogens.^32^ Clonal neoantigens were shown to elicit T cell immunoreactivity and sensitivity to blockade of the PD-1/PD-L1 axis.^33^ Based on these data, the pre-existence of CD8(+) T cells in the tumor (TILs) would be critical. More importantly, IHC evidence of negative regulation of tumor infiltrating lymphocytes (TIL) by the PD-1/PD-L1 axis, was correlated with clinical response to checkpoint blockade.^21^

As data continues to accumulate, a consensus is emerging that these immune modulations would likely be ineffective against tumors with low immunogenicity because the presence of tumor-specific lymphocytes is required for their clinical activity. Indeed, HER2 has been linked to immune resistance.^34^ This subset of patients, with “T-cell resistant” HER2(+) tumor cells and/or insufficient clonal frequency of tumor-specific T-cells, would likely not benefit from immune checkpoint blockade alone. The unique property of our HER2-BsAb to recruit T cells of any specificity and direct them against established tumors with relative insensitivity to the PD-1 immune checkpoint pathway is of interest, as it directly addresses the known limitations of immune checkpoint blockade. In fact, our preliminary *in vivo* data showed no additional benefit of PD-1 blockade to the HER2-BsAb therapeutic efficacy (data not shown), even though tumor PD-L1 expression was upregulated substantially following T cell infiltration (Fig. 4E).

When compared with the existing platforms that target HER2, HER2-BsAb offers advantages. Shalaby and colleagues described the development of a bispecific (Fab')_2_ antibody (anti-HER2 Fab' × anti-CD3 Fab'), through expressing each Fab' separately, and ligating the two together by chemical conjugation.^14^ More recently, Junttila and colleagues developed a heterodimeric bispecific IgG (anti-HER2 × anti-CD3) using “knob-and-hole” format.^16^ Both formats are monovalent binding to either HER2 or CD3, and substantially different both structurally and functionally when compared with our current HER2-BsAb for the following reasons: (1) The bivalent binding to HER2 is critical for the anti-proliferation capability, which is preserved in our construct (Fig. 1C) but not in those two monovalent systems, as demonstrated by Juntilla et al. who showed that the anti-proliferation capability of monovalent binding to HER2 (either heterodimeric bispecific IgG or trastuzumab-Fab) was 10-fold lower than bivalent trastuzumab.^16^ The dual mechanism (anti-proliferation plus T cell cytotoxicity) may create synergism and partly explains the potent efficacy of our BsAb *in vivo*. This may provide a salvage option for patients who progress on standard HER2-based therapies, or a replacement for trastuzumab given its dual anti-proliferative and T cell retargeting properties. (2) The bivalent binding to HER2 in our BsAb maintains high avidity (Fig. 1B) so as to maximize tumor binding, while the monovalent binding to HER2 (either heterodimeric bispecific IgG or trastuzumab-Fab) is 10-fold lower than trastuzumab.^16^ Higher avidity results in higher T-cell-dependent cell cytotoxicity, a phenomenon we and others have demonstrated in T-cell-engaging BsAb.^35^ Additionally, we hypothesize that the high avidity of our BsAb contributes to overcoming PD-1/PD-L1 checkpoints (Fig. 3), whereas the monovalent system by Juntilla's was shown to be inhibited by the PD-1/PD-L1 axis. (3) Our BsAb has the trastuzumab IgG backbone, preserving its pharmacologic advantages, while Shalaby's construct does not have FcR(n) affinity and should have much shorter serum half-life, and probably need to be administered as continuous infusion like Blinatumomab to be effective *in vivo*. 4) The other advantage is manufacturability: once CHO stable line is established, our BsAb can be produced in large scale and purified like normal IgG without significant aggregation despite prolonged incubation at 37°C, while chemical conjugates require more complicated syntheses and downstream processing—each Fab' expressed and purified separately, chemically modified, and then the two chemically conjugated and repurified. To ensure that a final product that is pure and chemically stable for direct clinical infusion is technically challenging and costly. Such chemically cross-linked reagents have only been feasible for *ex vivo* arming of T cells, but not for direct parenteral injections in the clinic.^36^

Our primary goal is to build a BsAb which has the bivalent binding to tumor targets (to preserve high avidity and/or anti-proliferation capability) and the monovalent binding to CD3 on effector T cells (to minimize spontaneous T cell activation in the absence of tumor targets). We surveyed several uniquely different bivalent formats, including chemical conjugation,^37^ dual-variable-domain (DVD), or attaching huOKT3 scFv to different positions in the IgG backbone (C-terminal of heavy chain or C-terminal of light chain),^38^ and found that the last option gave the best functionality. Although our BsAb has anti-CD3 scFv attached to both light chains, its reaction with CD3 on T cells was considered as functionally monovalent primarily for the following reasons: (1) Although our BsAb format contains two anti-CD3 scFvs positioned at the end of the light chains, these scFvs are oriented in geometrically opposed directions which restrict their ability to cooperatively bind to neighboring CD3 on T cells. This restricts the BsAb from binding bivalently and hence results in functional monovalency to CD3. We have previously shown in a different BsAb format that geometrical restriction of two anti-CD3 scFv can result in functionally monovalent binding to T cell and lower cytokine release.^35^ (2) The functional consequence of bivalent binding to CD3 on T cells is the triggering of spontaneous T cell activation, hence strong cytokine release in the absence of tumor targets. As shown in Fig. S2A, our BsAb only stimulated background cytokine release similar to that of the monovalent huOKT3 Fab, while bivalent huOKT3 IgG induced substantially more cytokines in the absence of tumor targets (left panel). However, in the presence of HER2(+) NCI-N87 tumor target, antitumor TH1 cytokines (TNFα and IFNγ) were released but only in the presence of BsAb (right panel), a format previously shown to induce immunologic synapse formation between the T cells and tumor targets.^20^ Furthermore, only bivalent huOKT3 IgG induced robust T cell proliferation, while our BsAb and monovalent huOKT3 Fab had negligible effects comparable to the T cells only control (Fig. S2B). In addition, the aglycosylation of the Fc removed both ADCC and most CMC functions, thereby further reducing cytokine release without affecting serum PK or compromising T cell activation.

CAR technology has rapidly accelerated investigations into HER2-directed gene-modified T cells in several clinical trials: NCT00902044 (for sarcoma), NCT00889954 (all HER2(+) cancers), NCT01109095 (GBM), NCT00924287 (metastatic cancer) and NCT01935843 (HER2(+) solid tumors). Toxicities from off target effects was initially concerning,^39^ although subsequent patients have been safely managed pharmacologically. There, the ability of T cells to overcome low levels of antigen expression was again observed. Osteosarcoma was a good example where the expression level has been controversial,^40^ and where CAR-modified T cells were highly efficient against locoregional and metastatic xenografts,^41^ and against osteosarcoma tumor initiating cells.^42^ Although the successful clinical application of CAR T cells has reassured many skeptics, there remain obstacles, including the necessity of cytoreductive chemotherapy before T cell infusion for meaningful clinical responses, logistics of cell harvest, processing, storage, transport and product release, T cell exhaustion^43^ and inadequate T cell persistence after infusion.

In summary, this report is the first demonstration of a successful IgG-scFv platform to engage T cells for HER2-directed immunotherapy. This BsAb for retargeting T cells is built with structural considerations for bivalency toward the target, and functionally monovalency toward CD3 on effector T cells, plus Fc aglycosylation for minimal spontaneous cytokine release. Its relative insensitivity to the PD-1/PD-L1 axis was novel. With the excellent antitumor activity both *in vitro* and *in vivo*, which is superior to trastuzumab counterpart, HER2-BsAb has considerable clinical potential.

## Materials and methods

### Cell lines

All cell lines were purchased from ATCC (Manassas Va) except: UM-SCC47 obtained from Dr Carey at the University of Michigan, SCC-90, PCI-30 and PCI-15B from Dr Robert Ferris at the University of Pittsburgh, SKOV3-luc from Dr Dmitry Pankov at MSK, 93-VU-147T and HeLa from Dr Luc Morris and UD-SCC2 from Henning Bier at Hals-Nasen-Ohrenklinik und Poliklinik. All cells were authenticated by short tandem repeat profiling using PowerPlex 1.2 System (Promega), and periodically tested for mycoplasma using a commercial kit (Lonza). The luciferase-labeled tumor cell lines MCF7-Luc were generated by retroviral infection with a SFG-GFLuc vector.

### HER2-BsAb design and expression in CHO-S cells

In the HER2-BsAb IgG-scFv format, VH was identical to that of trastuzumab IgG1, except N297A mutation in the Fc region was introduced to remove glycosylation, thereby depleting Fc function. The light chain was constructed by extending the trastuzumab IgG1 light chain with a C-terminal (G_4_S)_3_ linker followed by huOKT3 scFv. The DNA encoding both heavy chain and light chain was inserted into a mammalian expression vector, transfected into CHO-S cells, and stable clones of highest expression were selected. Supernatants were collected from shaker flasks and the HER2-BsAb was purified by protein A affinity chromatography. The other control BsAb, HER2-C825, was generated as described previously.^18^ HuOKT3 IgG1 was made using the same variable sequences as in huOKT3 scFv, and huOKT3 Fab was prepared from huOKT3 IgG1 using the Pierce Fab Preparation Kit (Thermo Scientific).

### Other antibodies and small molecules

Fluorophore-labeled HER2-BsAb was generated with the Zenon® Alexa Fluor® 488 Human IgG Labeling Kit from Life Technologies following the manufacturer's instructions. Pembrolizumab, cetuximab, trastuzumab, Erlotinib, Lapatinib and Neratinib were purchased from the MSKCC pharmacy. Small molecules were re-suspended in DMSO. The CD3, CD4, CD8 and CD56 antibodies were purchased from BD Biosciences (San Jose CA). The commercially available PE labeled PD-L1 specific mAb 10F.9G2 was purchased from BioLegend.

### Cell proliferation assays

For tumor cell proliferation, 5,000 tumor cells were plated using RPMI-1640 supplemented with 10% FBS in a 96-well plate for 36 h before being treated with kinase inhibitors or the antibodies at the specified concentration. Cell proliferation was determined using the cell counting WST-8 kit (Dojindo technologies) following the manufacturer's instructions and using the formula: % survival rate = (Sample−Background)/(Negative control−Background). Lapatinib was ground using a mortar and pestle and suspended in DMSO as described previously.^44^ To determine statistical significance, the results were analyzed using one-way ANOVA using Prism 6.0.

For T cell proliferation, naive T cells were purified from human PBMC using Pan T cell isolation kit (Miltenyi Biotec). 2 × 10^5^ purified T cells were mixed with different antibodies in 96-well cell culture plate to a final volume of 250 uL/well. T cells were cultured and maintained in RPMI-1640 supplemented with 10% FBS in 37°C for 6 d. T cell proliferation was quantitated using the WST-8 kit as described above.

### Cytotoxicity assays (^51^chromium release assay)

Cell cytotoxicity was assayed by ^51^Cr release as described previously,^20^ and EC_50_ was calculated using SigmaPlot software. Effector PBMC cells were purified from buffy coats purchased from the New York Blood Center. ATC were first purified from human PBMC using Pan T cell isolation kit, and then activated and expanded for approximately 14 d with CD3/CD28 Dynabeads (Invitrogen) according to manufacturer's protocol. For pre-incubation experiment, HER2-BsAb was pre-incubated with either ATCs (T cells pre-armed) or chromium-labeled tumor target cells (AU565 pre-targeted) for 30 min at RT, and unbound BsAb was washed off for two times before adding the other cells.

#### Cytokine release assay

Cytokine release was assayed as described previously,^35^ using naive T cells prepared as described above. T cells (200,000/well) were cultured with or without NCI-N87 tumor cells (10,000/well) for 24 h before supernatants being harvested for ELISA-based cytokine assay.

#### PD-1/PD-L1 expression

To overexpress PD-L1 in HEK293 cells, cells were cultured in DMEM (Cellgro) supplemented with 10% heat-inactivated FBS and Penicillin (100 IU/mL) and streptomycin (100 ug/mL). HEK293 cells were plated into six-well plates at 0.5 million cells/well with 2 mL fresh media the day before transfection. Transfection was done with 2.5 ug hPD-L1 plasmid DNA using Lipofectamine 2000 (Invitrogen) according to manufacturer's protocol. Cells were incubated at 37°C for 48 h before harvesting with 2 mM EDTA in PBS. 100–200 k cells were used for FACS analysis and the rest were used for the killing assays.

To induce PD-1 expression in ATCs, effector cells were incubated in a 3:1 ratio for 24 h with the HER2(+) breast carcinoma cell line HCC1954, after these target cells were incubated with HER2-BsAb at a concentration of 10 µg/mL for 30 min and excessive antibody was removed. Cells were harvested and used in cytotoxicity assays as described previously against the HEK293 cells or HCC1954 cells. For PD-1 blockade, PD-1-induced ATCs were pre-incubated with 10 ug/mL pembrolizumab for 30 min before adding to the well.

#### In vivo experiments

All animal procedures were performed in compliance with Institutional Animal Care and Use Committee (IACUC) guidelines. For *in vivo* therapy studies, BALB-*Rag2*^−/−^IL-2R-γc-KO (DKO) mice (derived from colony of Dr Mamoru Ito, CIEA, Kawasaki, Japan)^45,46^ were used. Four humanized mouse xenograft models were used: (1) iv tumor plus iv effector cells, (2) subcutaneous (sc) tumor plus sc effector cells, (3) sc tumor plus iv effector cells and (4) ip tumor plus ip/iv effector cells. Patient-derived xenografts (PDXs) were established from fresh surgical specimens with MSKCC IRB approval. Effector PBMC cells and ATCs were prepared as described above. Prior to every experimental procedure, PBMCs and ATCs were analyzed by FACS for their percentage of CD3, CD4, CD8 and CD56 cells to ensure consistency. Antibodies were injected i.v. or i.p. twice a week started 2 d before effectors cells for 3 to 6 weeks, given as i.v. 5–10 × 10^6^ PBMC/ATC per injection, once a week for 2 to 3 weeks. sc xenografts were created with tumor cells suspended in Matrigel (Corning Corp, Tewksbury MA) and implanted in the flank of DKO mice. Tumor size was measured using (1) hand-held TM900 scanner (Pieira, Brussels, BE), (2) Calipers or (3) Bioluminescence. Bioluminescence imaging was conducted using the Xenogen *In Vivo* Imaging System (IVIS) 200 (Caliper LifeSciences). Briefly, mice were injected i.v. with 0.1 mL solution of D-luciferin (Gold Biotechnology; 30 mg/mL stock in PBS). Images were collected 1 to 2 min after injection using the following parameters: a 10- to 60-sec exposure time, medium binning, and an 8 f/stop. Bioluminescence image analysis was performed using Living Image 2.6 (Caliper LifeSciences).

#### IHC staining

The immunohistochemical detection was performed at Molecular Cytology Core Facility of MSK using Discovery XT processor (Ventana Medical Systems). Paraffin-embedded tumor sections were deparaffinized with EZPrep buffer (Ventana Medical Systems), antigen retrieval was performed with CC1 buffer (Ventana Medical Systems) and sections were blocked for 30 min with Background Buster solution (Innovex). Anti-CD3 (DAKO, cat# A0452, 1.2 ug/mL), anti-HER2 (Enzo, cat# ALX-810–227, 5 ug/mL), and anti-PD-1 (Ventana, cat# 760–4895, 3.1 ug/mL) antibodies were applied and sections were incubated for 5 h, followed by 60 min incubation with biotinylated goat anti-rabbit IgG (Vector laboratories, cat# PK6101) for CD3 and HER2 antibodies, or biotinylated horse anti-mouse IgG (Vector Labs, cat# MKB-22258) for PD-1 antibodies at 1:200 dilution. The detection was performed with DAB detection kit (Ventana Medical Systems) according to manufacturer's instruction. Slides were counterstained with hematoxylin and coverslipped with Permount (Fisher Scientific). For PD-L1 staining, the sections were pre-treated with Leica Bond ER2 Buffer (Leica Biosystems) for 20 min at 100°C. The staining was done on Leica Bond RX (Leica Biosystems) with PD-L1 mouse monoclonal antibody (Cell Signaling, cat# 29122, 2.5 ug/mL) for 1 h on Leica Protocol F. All images were captured from tumor sections using Nikon ECLIPSE Ni-U microscope and NIS-Elements 4.0 imaging software.

### Statistics

Differences between samples indicated in the figures were tested for significance by one-way ANOVA using Prism 6.0, and *p* < 0.05 was considered statistically significant.

## Supplementary Material

KONI_A_1267891_s02.pdf
