# Supplementary material for: Overcoming resistance to HER2-targeted therapy with a novel HER2/CD3 bispecific antibody
Source: Oncoimmunology. 2017 Mar 10;6(3):e1267891. doi: 10.1080/2162402X.2016.1267891 (PMC5384386; doi:10.1080/2162402X.2016.1267891)
Supplement: KONI_A_1267891_s02.pdf [file koni-06-03-1267891-s001.pdf]

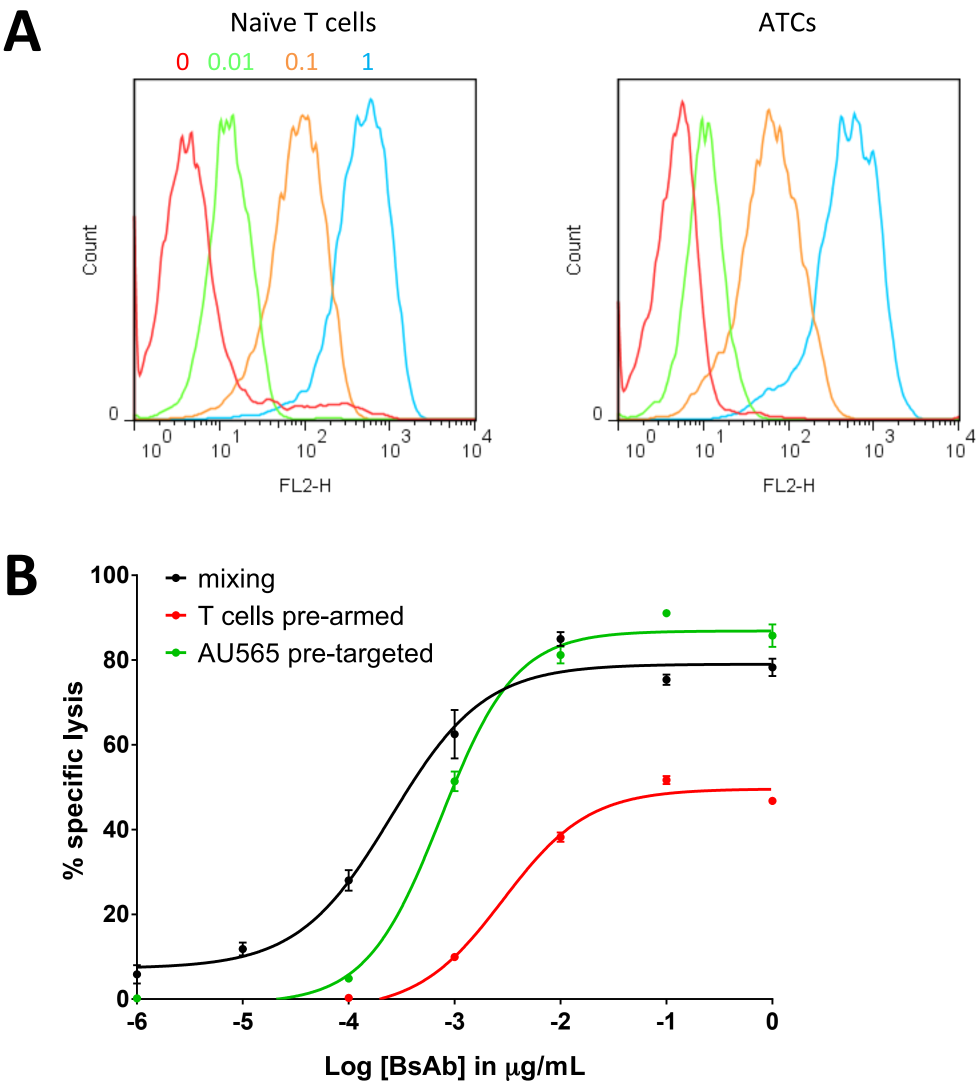

Supplemental Figure S1. HER2-BsAb binding to T cells and redirecting T-cell killing. (**A**) FACS histograms of HER2-BsAb binding to naïve T cells purified from fresh PBMC (left panel) or ATCs (right panel). Concentrations of BsAbs (μg/10^6 cells) were recorded on the top of the left histogram, and Rituxan was used as negative control (mean fluorescence intensity set at 5). (**B**) HER2-BsAb redirected T-cell killing of HER2(+) AU565 breast cancer cells by 4 hr ^{51}Cr release assay. BsAb was either mixed directly with T cells and AU565 together (mixing), or pre-incubated with T cells/target first (T cells pre-armed or AU565 pre-targeted), and unbound BsAb washed off before adding the other cells. ATC-to-target ratio was 10:1. Data points are shown as Mean ± SEM.

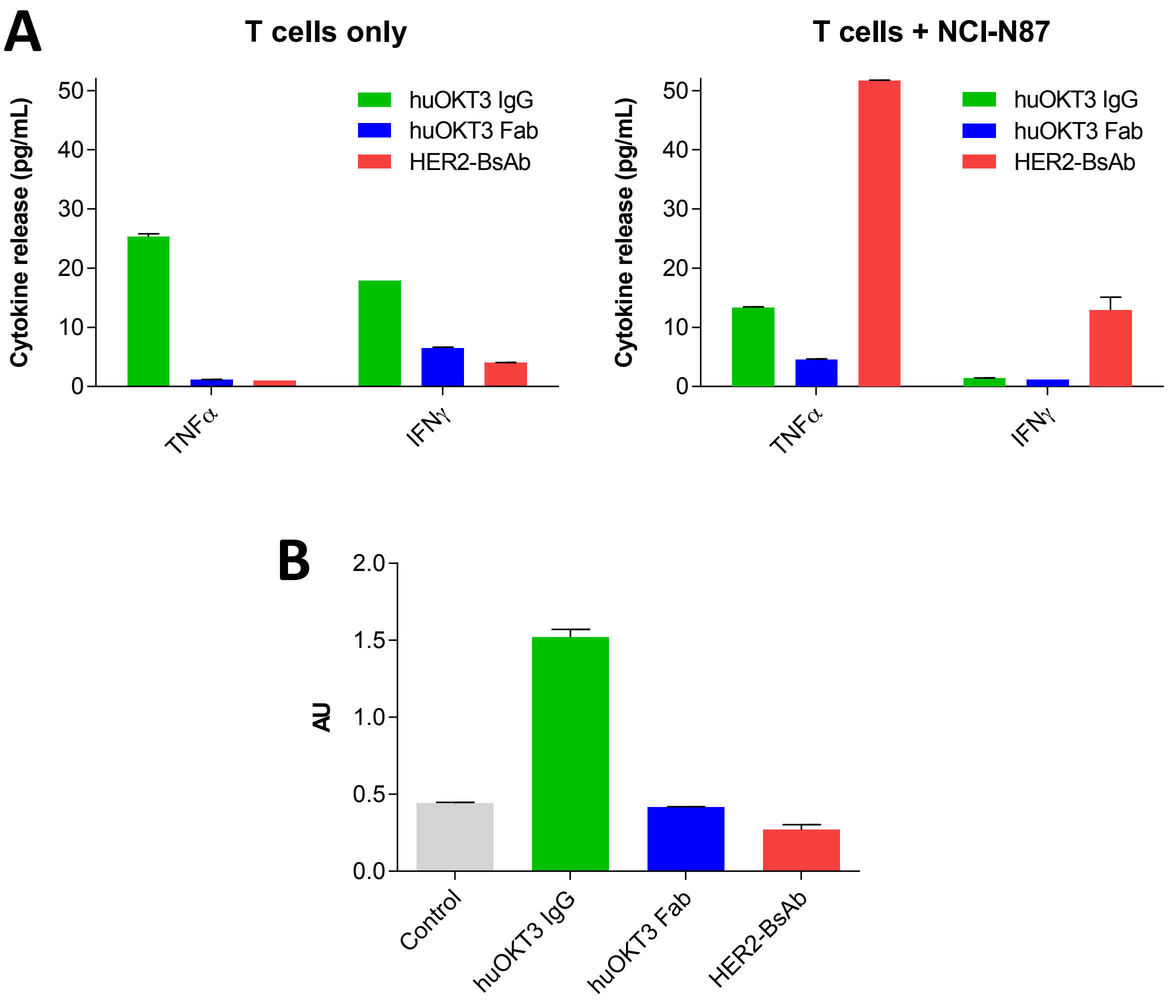

Supplemental Figure S2. HER2-BsAb binding to CD3 on T cells was functionally monovalent. (**A**) Cytokine release from naïve T cells induced by 16.7 nM HER2-BsAb when compared to bivalent huOKT3 IgG and monovalent huOKT3 Fab, in the absence (left panel) or presence (right panel) of HER2(+) NCI-N87 gastric tumor cells. Cytokine release level below detection level was assigned as 1 pg/mL. (**B**) T cell proliferation stimulated by 67 nM of the related antibodies, in the absence of tumor targets. T cells only (Control) as the negative control. OD reading at 450 nm (AU) was shown. All data points are shown as Mean + SD.
